# Kojic acid reverses LPS-induced neuroinflammation and cognitive impairment by regulating the TLR4/NF-κB signaling pathway

**DOI:** 10.3389/fphar.2024.1443552

**Published:** 2024-08-09

**Authors:** Waqar Ali, Kyonghwan Choe, Jun Sung Park, Riaz Ahmad, Hyun Young Park, Min Hwa Kang, Tae Ju Park, Myeong Ok Kim

**Affiliations:** ^1^ Division of Life Science and Applied Life Science (BK21 FOUR), College of Natural Sciences, Gyeongsang National University, Jinju, Republic of Korea; ^2^ Department of Psychiatry and Neuropsychology, School for Mental Health and Neuroscience (MHeNs), Maastricht University, Mastricht, Netherlands; ^3^ Department of Pediatrics, Maastricht University Medical Center (MUMC+), Maastricht, Netherlands; ^4^ Haemato-oncology/Systems Medicine Group, Paul O’Gorman Leukaemia Research Centre, Institute of Cancer Sciences, College of Medical, Veterinary and Life Sciences (MVLS), University of Glasgow, Glasgow, United Kingdom; ^5^ Alz-Dementia Korea Co., Jinju, Republic of Korea

**Keywords:** LPS, neuroinflammation, oxidative stress, neurodegeneration, cognitive impairment, kojic acid

## Abstract

Intense neuroinflammation contributes to neurodegenerative diseases, such as Alzheimer’s disease and Parkinson’s disease. Lipopolysaccharides (LPSs) are an integral part of the cell wall of Gram-negative bacteria that act as pathogen-associated molecular patterns (PAMPs) and potentially activate the central nervous system’s (CNS) immune system. Microglial cells are the local macrophages of the CNS and have the potential to induce and control neuroinflammation. This study aims to evaluate the anti-inflammatory and antioxidant effect of kojic acid against the toxic effects of LPSs, such as neuroinflammation-induced neurodegeneration and cognitive decline. The C57BL/6N mice were subjected to LPS injection for 2 weeks on alternate days (each mouse received 0.25 mg/kg/i.p. for a total of seven doses), and kojic acid was administered orally for 3 weeks consecutively (50 mg/kg/mouse, p. o). Bacterial endotoxins, or LPSs, are directly attached to TLR4 surface receptors of microglia and astrocytes and alter the cellular metabolism of immune cells. Intraperitoneal injection of LPS triggers the toll-like receptor 4 (TLR4), phospho-nuclear factor kappa B (p-NFκB), and phospho-c-Jun n-terminal kinase (p-JNK) protein expressions in the LPS-treated group, but these expression levels were significantly downregulated in the LPS + KA-treated mice brains. Prolong neuroinflammation leads to the generation of reactive oxygen species (ROS) followed by a decrease in nuclear factor erythroid-2-related factor 2 (Nrf2) and the enzyme hemeoxygenase 1 (HO-1) expression in LPS-subjected mouse brains. Interestingly, the levels of both Nrf-2 and HO-1 increased in the LPS + KA-treated mice group. In addition, kojic acid inhibited LPS-induced TNF-α and IL-1β production in mouse brains. These results indicated that kojic acid may suppress LPS-induced neuroinflammation and oxidative stress in male wild-type mice brains (in both the cortex and the hippocampus) by regulating the TLR4/NF-κB signaling pathway.

## 1 Introduction

Neuroinflammation is mainly involved in the pathogenesis of neurodegenerative diseases, such as Alzheimer's disease (AD) and Parkinson’s disease (PD) (Leng and Edison, 2021; Wetering et al., 2024). Innate immunity plays an important role in managing central nervous system (CNS) insults. Therefore, a short-term upregulation in inflammatory response is natural and has no detrimental effects on the functions of CNS. However, chronic neuroinflammation may lead to neurological diseases such as AD and PD (McGeer and McGeer, 2004; Calsolaro and Edison, 2016). The CNS consists of glial cells, including microglia and astrocytes, which act as an immune system against exogenous and endogenous pathogens and regulate normal brain functions (Tyrtyshnaia et al., 2020). Various factors, along with systemic inflammation, mimic the brain's glial cells. As a result, there will be a release of inflammatory mediators, which also cause neurodegenerative diseases (Chiang et al., 2023). Neuroinflammation has four different stereotyped mechanisms, such as increased levels of chemo-toxic substances (reactive oxygen species (ROS), reactive nitrogen species (RNS), and NO) (Chitnis and Weiner, 2017), activation and proliferation of glial cells (microglia and astrocytes) (Radenovic et al., 2020), peripheral immune cell infiltration from general circulation (monocytes, macrophages, and T-lymphocytes) (Dansokho et al., 2016), and neuronal cell death due to neurotoxic substances (Skaper et al., 2018; Moyse et al., 2022). However, the glial and immune cells contribute to neuroinflammation by i) sensing “harmful signaling” from pathogens and damaged tissue, ii) phagocytosis of neuronal lesions, and iii) synthesis of chemical messenger and accumulation of other effector cells (DiSabato et al., 2016).

Different experimental animal models have been developed, such as amyloid beta (Aβ), D-galactose, and lipopolysaccharide (LPS). These models potentially lead to neuroinflammation and elevated levels of ROS. The lipopolysaccharides (LPSs) are bacterial endotoxins that activate both the neuroinflammatory and oxidative stress pathways and lead to neurodegenerative-like pathology (Noworyta-Sokołowska et al., 2013). LPSs accumulate in vital organs of the living body, such as kidneys, lungs, liver, and, more specifically, in the brain. In the brain, LPSs cause neurotoxicity, neuro-inflammation, oxidative stress, synaptic dysfunction, and finally, neurodegeneration (Khan et al., 2018). Peripheral signals via the vagal nerve system induce neuroinflammation and lead to the release of inflammatory mediators such as TNF-α, IL-1β, and prostaglandins (Pu et al., 2022).

A prolonged neuronal inflammatory response with bacterial toxins (LPSs) contributes to CNS malfunction (Gu et al., 2021). The CNS malfunctions are followed by neuronal inflammation and memory impairment. Neuroinflammation is triggered by neuronal injury and tissue infection, which promote different biochemical cascades. These cascading pathways increase the activation of pro-inflammatory cytokines, microglia activation, and intense oxidative stress. Oxidative stress leads to the generation and localization of ROS both at the level of cells and tissues (Ali and Kim, 2015). Elevated oxidative stress exaggerates neuronal cell loss and memory dysfunction (Luo et al., 2018). The nuclear factor-erythroid 2 (Nrf2) and heme oxygenase-1 (HO-1) both act as an antioxidant against oxidative stress (Ma, 2013). Normally, Nrf2 is located in the cytoplasm with kelch-like ECH-associated protein 1 (Keap-1) in an inactive form (Vizuete et al., 2022). Several biochemical factors dissociate Nrf2 from Keap-1 and translocate it from the cytoplasm to the nucleus, where other molecular pathways are initiated (Xu et al., 2011). Excessive generation of ROS may lead to the failure of the antioxidant defense mechanism (Ikram et al., 2019). Disturbances in the homeostasis of antioxidant proteins (i.e., Nrf2 and HO-1) (Woodburn et al., 2021) may increase vulnerability to neurodegenerative diseases, such as AD and PD.

Microglial cells have the potential to induce and control neuroinflammation. Thus, inhibiting microglial cells plays a keen role in controlling neuroinflammation and providing an effective therapeutic approach for AD-like pathology (Ali et al., 2020). LPS-induced neuroinflammation mimics the immune system, which causes further neurodegeneration (Peng et al., 2007). The regulation of immunological responses is critical because most neurological diseases are associated with chronic activation of immune cells (Zhao et al., 2019).

Toll-like receptor-4 (TLR4) is a transmembrane pattern recognition receptor (PRR) (Adesso et al., 2018) of the innate immune system. TLR4 is present on the surface of many types of cells, such as macrophages, microglial cells, astrocytes, and other neuronal cells (Gilhus and Deuschl, 2019). TLR4-receptor complexation with LPS promotes AD-like pathology, and blocking of TLR4-receptor may reverse NF-κB (nuclear factor-κB p65) upregulation and downregulation of inflammatory biomarkers both in the cortex and hippocampus of rodent brain (Lu et al., 2008). Accumulated neuroinflammation and induced oxidative stress may trigger synaptic plasticity, which causes alteration in neuronal communication (Lin et al., 2019) and, finally, memory loss (Sheppard et al., 2019). The main markers, including synaptosomal-associated protein 23 (SNAP-23) and postsynaptic density protein 95 (PSD-95), are involved in synaptic dysfunction and neurodegeneration (Kolmogorova and Ismail, 2021).

Several natural substances have shown greater efficacy as anti-inflammatories and antioxidants. Kojic acid (KA, 5-hydroxy-2-hydroxymethyl-1–4-pyrone) is a natural biological active compound isolated from fungus *Aspergillus* species (de Caldas Felipe et al., 2023). Kojic acid is widely used in cosmaceuticals to reduce skin hyperpigmentation and aging (Saeedi et al., 2019). KA also has both antibiotic and antifungal properties (Rodrigues et al., 2014). Kojic acid is a potent antioxidant and effectively minimizes the risks of oxidative stress-induced diseases (Moon et al., 2001). Similarly, kojic acid exerts an anti-inflammatory effect and inhibits activation of phosphorylated nuclear factor-κB (p-NFκB) in various types of cells, such as HaCaT and SCC-13 cells, and in LPS-treated RAW264.7 macrophages (Lee et al., 2019). Kojic acid is also used to treat different peripheral inflammation diseases in experimental mice (Zilles et al., 2022).

Herein, by using Western blot, immunofluorescence studies, and some biochemical assays, we investigated whether the oral administration of kojic acid might alleviate LPS-induced TLR4-mediated neuroinflammation, oxidative stress, and memory and cognitive impairment *in vivo*.

## 2 Materials and methods

### 2.1 Chemicals

Kojic acid (KA), LPS, and 2,7-dichlorodihydrofluorescein diacetate (DCFH-DA) were acquired from Sigma-Aldrich Chemical Co. (St. Louis, MO, United States).

### 2.2 Antibodies

Table 1 reports the list of antibodies used in this study.

**TABLE 1:** Antibodies used in the Western blot and immunofluorescence testing.

Antibody	Source	Manufacturer	Ref.	Application	Dilution (μL)
TLR4	Mouse	Santa Cruz	Sc-293072	Western blot/Confocal	1:1000/1:100
Iba-1	Rabbit	Cell Signaling, United States of America	17,198	Western blot/Confocal	1:1000/1:100
GFAP	Mouse	Santa Cruz	Sc-33673	Western blot	1:1000
p-JNK	Mouse	Santa Cruz	Sc-6254	Western blot	1:1000
p-NF- KB	Mouse	Santa Cruz	Sc-136548	Western blot	1:1000
TNF-α	Mouse	Santa Cruz	Sc-52746	Western blot/Confocal	1:1000/1:100
IL-1β	Mouse	Santa Cruz	Sc-32294	Western blot	1:1000
Nrf2	Rabbit	Cell Signaling, United States of America	12721S	Western blot/Confocal	1:1000/1:100
HO-1	Mouse	Santa Cruz	Sc-136961	Western blot	1:1000
PSD-95	Mouse	Santa Cruz	Sc-71933	Western blot/Confocal	1:1000
SNAP-23	Mouse	Santa Cruz	Sc −374215	Western blot	1:1000
β- Actin	Mouse	Santa Cruz	Sc-47778	Western blot	1:1000

All the secondary antibodies were diluted in 1× TBST, with a concentration of 1:10000 μL.

### 2.3 Experimental animals

Wild-type C57BL/6N 8-week male mice (total mice n = 32, eight mice per group) weighing 28–30 g were acquired from Samtako Bio, Osan, South Korea. We prefer mostly male mice over female mice to avoid any potential variation caused by sex differences and the female estrous cycle. The female mice exhibit different anatomical structures, physiology, and certain behaviors. The estrous cycle increases variation in female behavior relative to male mice (Tsao et al., 2023). All the male mice were handled carefully and followed the approved guidelines (Approval ID: 125, Code: GNU-200331-M0020) of the Institutional Animal Care and Use Committee (IACUC) of the Division of Applied Life Science, Gyeongsang National University, South Korea. The mice were acclimated for 7 days in an animal house, maintaining the temperature at 20◦C ± 2°C, the humidity at 40% ± 10%, a 12-h light/dark cycle, and fed with normal pellet food and water *ad libitum*.

### 2.4 Mice grouping and treatment

**FIGURE 1:**
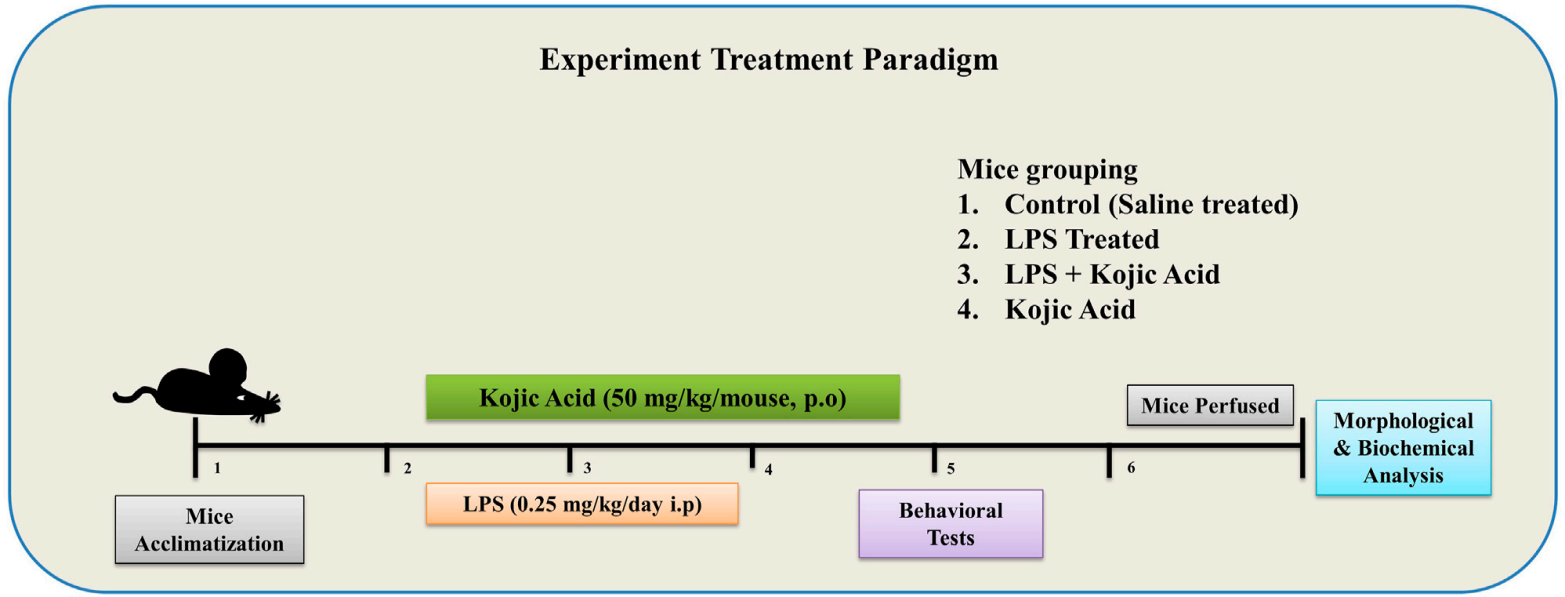
Schematic diagram of the experimental design to examine the use of kojic acid against LPS-induced neuroinflammation and oxidative stress-mediated neurodegeneration.

The experimental animals were divided into four groups (n = 8 per group; in each group, n = 4 for WB and n = 4 for IHC): the control group (injected i.p. 0.9% NaCl), the LPS-injected group (injected i.p. LPS 0.25 mg/kg/day), the LPS + Kojic acid group (LPS + KA, 50 mg/kg/day) and the kojic-acid-only group (50 mg/kg/day). LPS was injected i.p. for 2 weeks on every alternate day for a total of seven doses (Badshah et al., 2019), and KA was given orally (P.O.) for 3 weeks (Khan et al., 2021) (2 weeks along with LPS injection and 1 week post-LPS injection) through curved oral gavage (Figure 1).

### 2.5 Preparation of kojic acid for oral administration

KA was dissolved in a small amount of normal saline (0.9%), and then the final volume was adjusted by adding more normal saline. The dissolved KA was then poured into an amber glass bottle and stored at 4°C. The dissolved drug was ready for administration.

### 2.6 Behavioral studies

The Morris water maze (MWM) and Y-maze tests were both performed on all mice (each group consists of n = 8 mice) to examine the behavior of all experimental groups. The Morris water maze is usually used to measure the memory and learning capability of mice. The MWM is circular shaped, 100 cm in diameter and 35 cm in height, and filled with water (23°C ± 1°C) to a depth of 15.5 cm containing non-toxic white-colored paint. A transparent hidden platform measuring 4.5 cm in diameter and 14.5 cm in height was slightly immersed at the mid-point of any quadrant. All the mice were individually placed in one quadrant and allowed to search for the hidden platform. Each mouse individually participated in four trials (freely swimming for 1 min) for 5 consecutive days to measure the escape latency from the water maze (finding an immersed hidden platform).5° On the next day, escape latency and probe tests were performed, and the hidden platform was removed. Then, the time spent by each mouse in a specific quadrant and the number of zones explored were measured.

Another Y-maze setup was used for spatial memory, consisting of three arms arranged in a Y-shape. The dimensions of each arm of the Y-maze setup were 50 cm length, 20 cm height, and 10 cm width, with a camera sensor attached on the top. Each mouse was individually placed in the center of the maze and allowed to move freely for 8 min. The number of entries into each arm is sensed by the attached sensor. The successive entry of mice in each arm in overlapping triplets was considered a spontaneous alternation. The percentage (%) alternation behavior is measured as successive triplet sets of entries into different arms/total number of arm entries × 100. The increase in the percentage of spontaneous behavior shows improved cognitive dysfunction.

### 2.7 Immunoblotting

The mice were then anesthetized by ketamine and xylazine according to the protocol previously described (Ahmad et al., 2022). The cortex and hippocampus from the mice brain were separated and homogenized with PRO-PREPTM extraction solution (iNtRON Biotechnology, Inc., Sungnam, South Korea) and centrifuged (13,000 RPM for 25 min at 4°C). After centrifugation, the supernatant was collected and kept at −70°C. The protein concentration was measured through the amplification of a Bio-Rad assay kit. Protein samples were electrophoresed on 10%/12.5% sodium dodecyl sulfate (SDS-PAGE GEL) (Merck KGaA, Darmstadt, Germany) and transferred to a polyvinylidene fluoride (PVDF) membrane (Immobilon-PSQ, transfer membrane, Merck Millipore, Burlington, MA, USA). The PVDF membranes were blocked in 5% skim milk for at least 1 h at room temperature (25°C). The PVDF was incubated with primary antibodies for 24 h diluted in 1X Tris-Buffered saline and 0.1% Tween^®^20 Detergent (1 × TBST). The next day, the PVDF was washed with 1 × TBST (three times for 10 min). Then, the membrane was incubated with secondary antibodies of appropriate source (anti-rabbit/anti-mouse, diluted in 1 × TBST) for 2 h at room temperature. The membrane was washed with 1 × TBST and subjected to an enhanced chemiluminescence (ECL) reagent (ATTO Corporation, Tokyo, Japan). The expression of protein bands was captured on X-ray film in a darkroom. The Western blot results were analyzed using ImageJ (v. 1.50, NIH, Bethesda, MD, USA) to compare with the control group. The β-actin was used as a loading control, and all the data were presented graphically using GraphPad Prism v8 software (GraphPad Software, San Diego, CA, United States).

### 2.8 Tissue sampling for immunofluorescence analysis

First, the mice were anesthetized with ketamine and xylazine and perfused with 0.1 M phosphate-buffered saline (PBS) and 4% neutral-buffered formalin. The whole brain was separated and blocked with optimal cutting temperature (OCT) compound. The brain was sliced (14 μm) using a microtome (Leica CM 1860 UV, Burladingen, Germany). The sections were thaw-mounted on gelatin-coated slides (Fisher, Rockford, IL, United States) and stored at −70°C.

### 2.9 Immunofluorescence staining

Immunofluorescence staining was performed, as reported previously (Rehman et al., 2019). All the slides were kept in a fume hood overnight at room temperature to dry. On the next day, the slides were washed with 0.1 M PBS for 10 min (two times). The slides were then incubated with proteinase K for 6 min and blocked for 1 h with a blocking solution containing 2% normal goat serum (rabbit/mouse) and 0.1% Triton X-100 in 0.1 M PBS. The slides were then incubated overnight at 4°C in primary antibodies. On the next day, the brain section was washed with 0.1 M PBS and incubated with secondary tetramethylrhodamine (TRITC)/fluorescein isothiocyanate (FITC)-labeled antibodies for 2 h. Each slide was rinsed with 0.1 M PBS for 6 min (two times). Finally, a few drops of 4, 6-diamidino-2-phenylindole dihydrochloride (DAPI) were applied for routine nuclear staining, and the slides were covered with coverslips. At this point, the slides were ready to be examined under a confocal laser microscope (FluoView FV1000 MPE, Olympus, Tokyo, Japan). The data were analyzed through the ImageJ analysis program. The image background was optimized according to the threshold intensity, and the immunofluorescence intensity, analyzed at the same threshold intensity for all groups, was expressed as the relative integrated density of the samples relative to the control.

### 2.10 Reactive oxygen species (ROS) assay

A ROS assay based on the conversion of 2,7-dichlorodihydrofluorescein diacetate (DCFH-DA) to 2,7-dichlorofluorescein (DCF) was performed. Homogenates of all groups of brains were taken and diluted in Lock’s buffer at a ratio of 1:20 (yield concentration of 2.5 mg tissue/500 µL). All the reactants, such as 0.2 mL of homogenate and 10 mL of DCFH-DA (5 mM), were added to Lock’s buffer (1 mL; pH 7.4) and left at room temperature for 15 min. The conversion of DCFH-DA to DCF was evaluated through a spectrofluorometer (Promega, Fitchburg, WI, USA) with emission at 530 nm and excitation at 484 nm. Blank samples were used to identify and remove the background signals.

### 2.11 Lipid peroxidation (LPO) assay

A lipid peroxidation (LPO) assay is an indicator of oxidative stress. A specific LPO assay kit was utilized (BioVision, San Francesco, CA, USA, Cat#739-100) in order to measure the level of free malondialdehyde (MDA) in the mice brain homogenates. The assay was carried out according to the manufacturer’s recommendations. The mice brains were homogenized in 300 μL of the MDA lysis buffer along with 3 μL BHT and then centrifuged (13,000/10 rpm). Approximately 10 mg of protein was precipitated from each brain sample in 150 μL distilled water +3 μL BHT, adding 1 mL of 2 N perchloric acid, vortexing, and again centrifuging to isolate the precipitated protein. The supernatant from each sample was added to a 96-well plate, and the absorbance (at 532 nm) was read through a microplate analyzer. The total MDA value was measured as nmol/mg of protein in each brain homogenate (cortex and hippocampus) (Ali et al., 2018).

### 2.12 Nissl staining

The Nissl staining of mice brains was performed as reported previously (Ali et al., 2015). The slides containing brain sections were washed for 15 min (2 times) in 0.01 M PBS. The 0.5% cresyl violet staining solution was prepared, and a few drops of glacial acetic acid were added. The slides were then incubated in 0.5% cresyl violet solution for 10–15 min at room temperature. After incubation, the slides were rinsed with distilled water and dehydrated with different grades of ethanol solution (70%, 95%, and 100%). The slides were immersed in a xylene mount with non-fluorescing media, and a coverslip was applied. Different regions were analyzed using the ImageJ analysis program.

### 2.13 Statistical analysis

The Western blot and immunofluorescence images were quantified using ImageJ software. The total data are presented as the mean ± SEM for the four independent experiments and analyzed using Prism v8 software (GraphPad Software, In., San Diego, CA, US). One-way ANOVA with Tukey’s post hoc testing was used to plot comparisons between groups. P-values less than 0.05 were considered to be statistically significant. The symbol # indicates a significant difference from the saline-injected group, while the symbol * indicates a significant difference from the LPS-injected group. Significance: ^#^ *p* ≤ 0.05, ^##^ *p* ≤ 0.01, and ^###^ *p* ≤ 0.001; *p ≤ 0.05, **p ≤ 0.01, and ***p ≤ 0.001.

## 3 Results

### 3.1 Kojic acid inhibited LPS-induced activated TLR4, astrocytes, and microglial markers in the mouse brain

**FIGURE 2:**
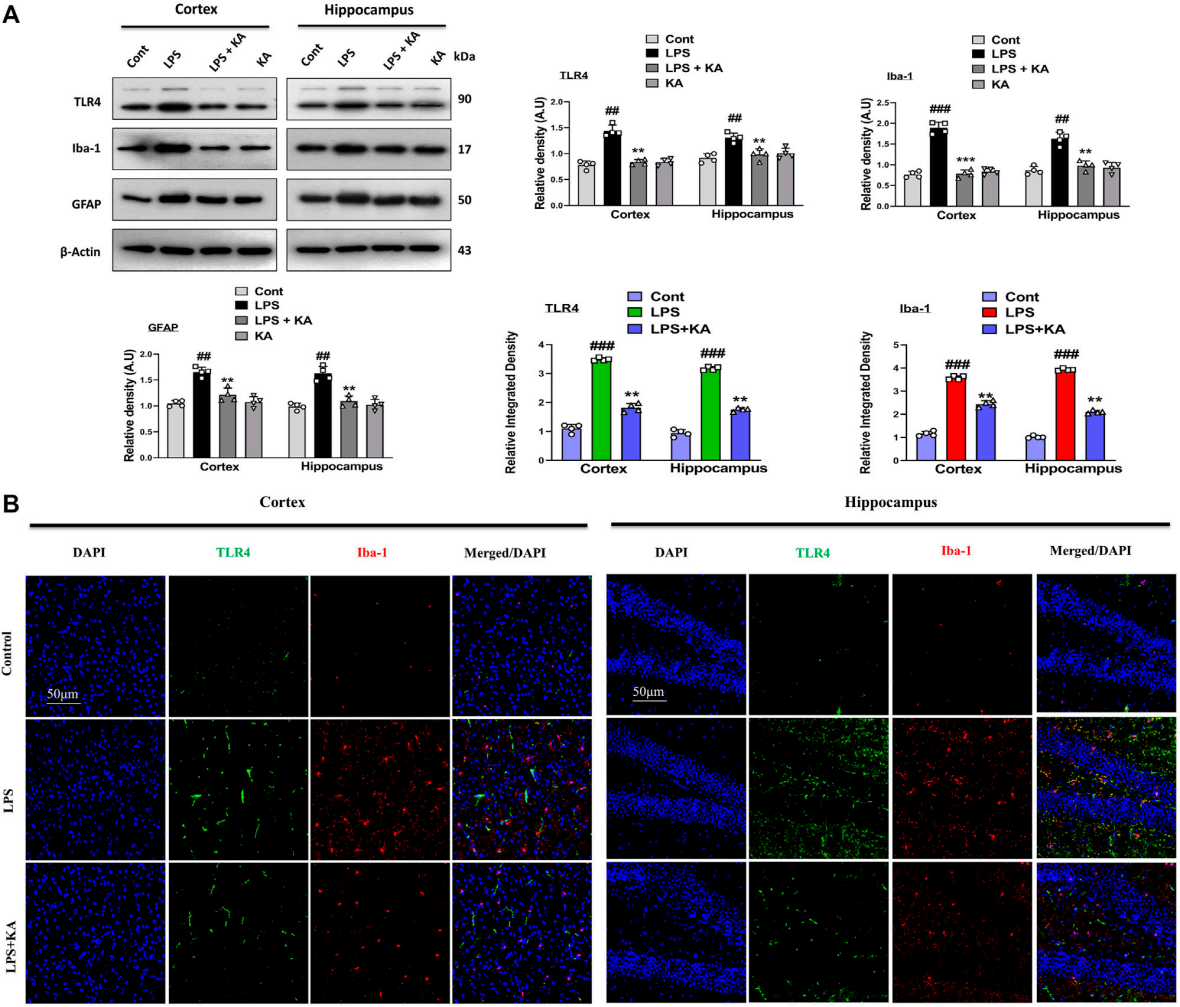
Effect of kojic acid versus LPS-induced TLR4, Iba-1, and GFAP in the mouse brain. **(A)** Immunoblot analysis of TLR4, Iba-1, and GFAP with respective bar graph (n = 4). **(B)** Immunofluorescence images of co-localized reactivity of TLR4 and Iba-1 in the mouse cortex and hippocampus (n = 4). The density values are relative to those of the control group and expressed in arbitrary units (AU), magnification ×10; scale bar 50 µm. All the data were measured in mean ± S.E.M. ^#^ significantly different from the saline-injected group, * significantly different from the LPS-injected group. Significance: ^#^ *p* ≤ 0.05, ^##^ *p* ≤ 0.01, and ^###^ *p* ≤ 0.001; *p ≤ 0.05, **p ≤ 0.01, and ***p ≤ 0.001.

LPS accumulation in the brain activates glial cells, which initiate inflammatory pathways. TLR4 is the glial cells' surface receptor response to inflammatory cascades. We examined the TLR4, ionized calcium-binding molecule 1 (Iba-1), and glial fibrillary acid protein (GFAP) levels in the cortex and hippocampus of the brain. These biomarkers were elevated in the case of chronic inflammation, followed by disease states. Our Western blot result confirmed enhanced expression levels of TLR4, Iba-1, and GFAP in the LPS-treated mouse group brains, and these levels were significantly lower in the LPS + KA co-treated group (Figure 2A). In addition, we performed co-staining of TLR4 and Iba-1, which also showed an increase in fluorescence intensity in the LPS-injected mice brain sections. Interestingly, these elevated fluorescence levels were decreased in the LPS + KA co-treated mice brains, which showed the possible anti-inflammatory activity of kojic acid against LPS-induced inflammation (Figure 2B).

### 3.2 Kojic acid regulates the expression of p-JNK and its downstream targets in LPS-treated mouse brains

**FIGURE 3:**
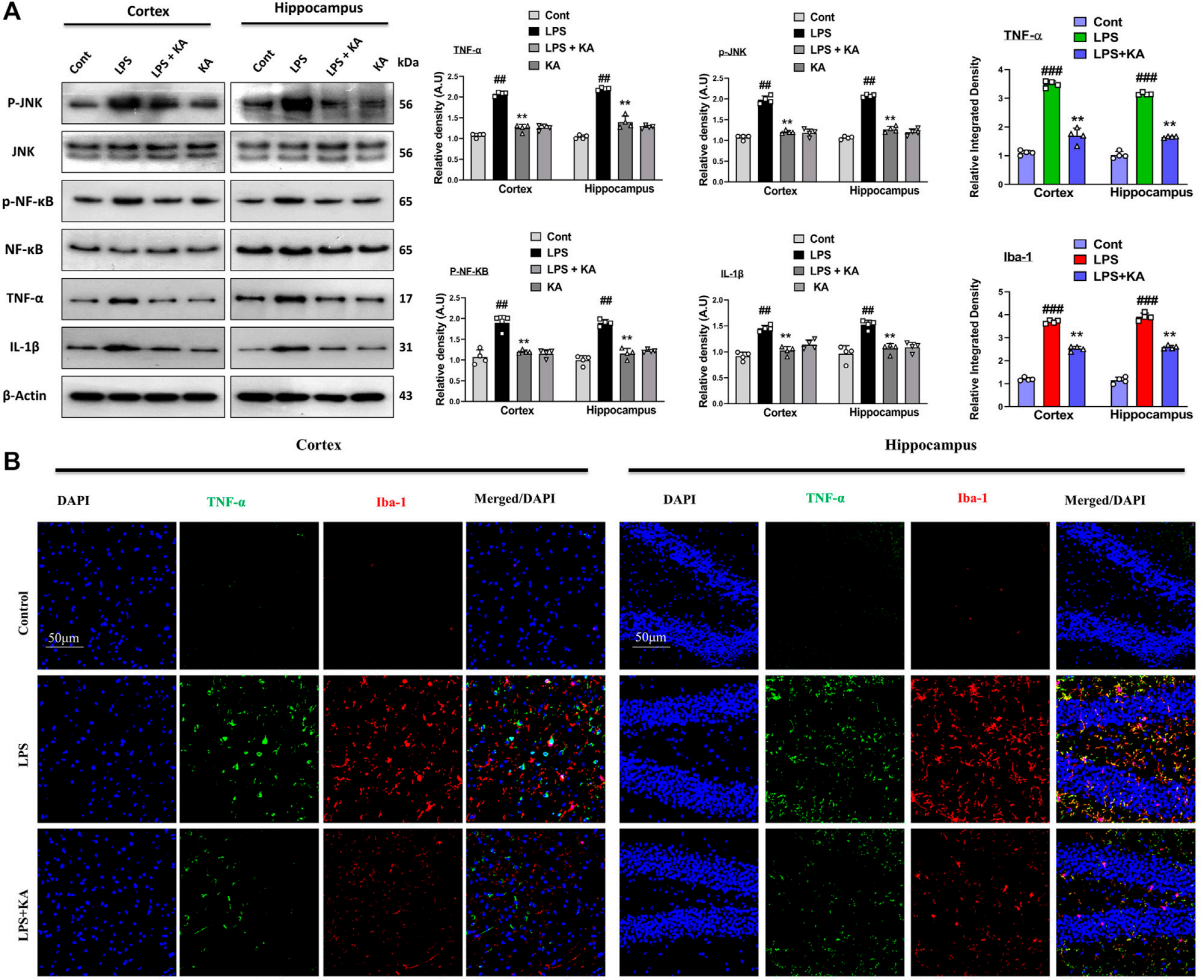
**(A)** Immunoblot analysis p-JNK, p-NF-κB, TNF-α, and IL-1β in experimental rodent groups with respective bar graphs (n = 4). **(B)** Immunofluorescence images of the co-localized reactivity of TNF-α and Iba-1 in the mouse cortex and hippocampus (n = 4) with respective bar graphs. Magnification ×10; scale bar 50 µm. All the data were measured in mean ± S.E.M. ^#^ significantly different from the saline-injected group, * significantly different from the LPS-injected group. Significance: ^#^ *p* ≤ 0.05, ^##^ *p* ≤ 0.01, and ^###^ *p* ≤ 0.001; *p ≤ 0.05, **p ≤ 0.01, and ***p ≤ 0.001.

To evaluate the expression of p-JNK and its downstream targets in the LPS-treated mouse brains, we performed Western blotting for phospho-c-Jun-N-terminal kinase (p-JNK), p-NF-κB, and tissue necrosis factor (TNF)-α in the brains of the experimental groups. Our results showed significantly increased expression levels of various inflammatory biomarkers (i.e., p-JNK, p-NF-κB, TNF-α, and IL-1β) in the LPS-subjected group compared to the saline-injected control group. Notably, these markers were significantly reduced in the LPS + KA-treated group (Figure 3A). These effects were further confirmed by the immunofluorescence results, which showed an enhanced expression of TNF-α along with Iba-1 in the cortices and the hippocampi of LPS-treated mice compared to the control group; interestingly, these biomarkers were significantly reduced in the LPS + KA-treated group, as shown in Figure 3B.

### 3.3 Kojic acid prevents LPS-induced oxidative stress in the mouse brain

**FIGURE 4:**
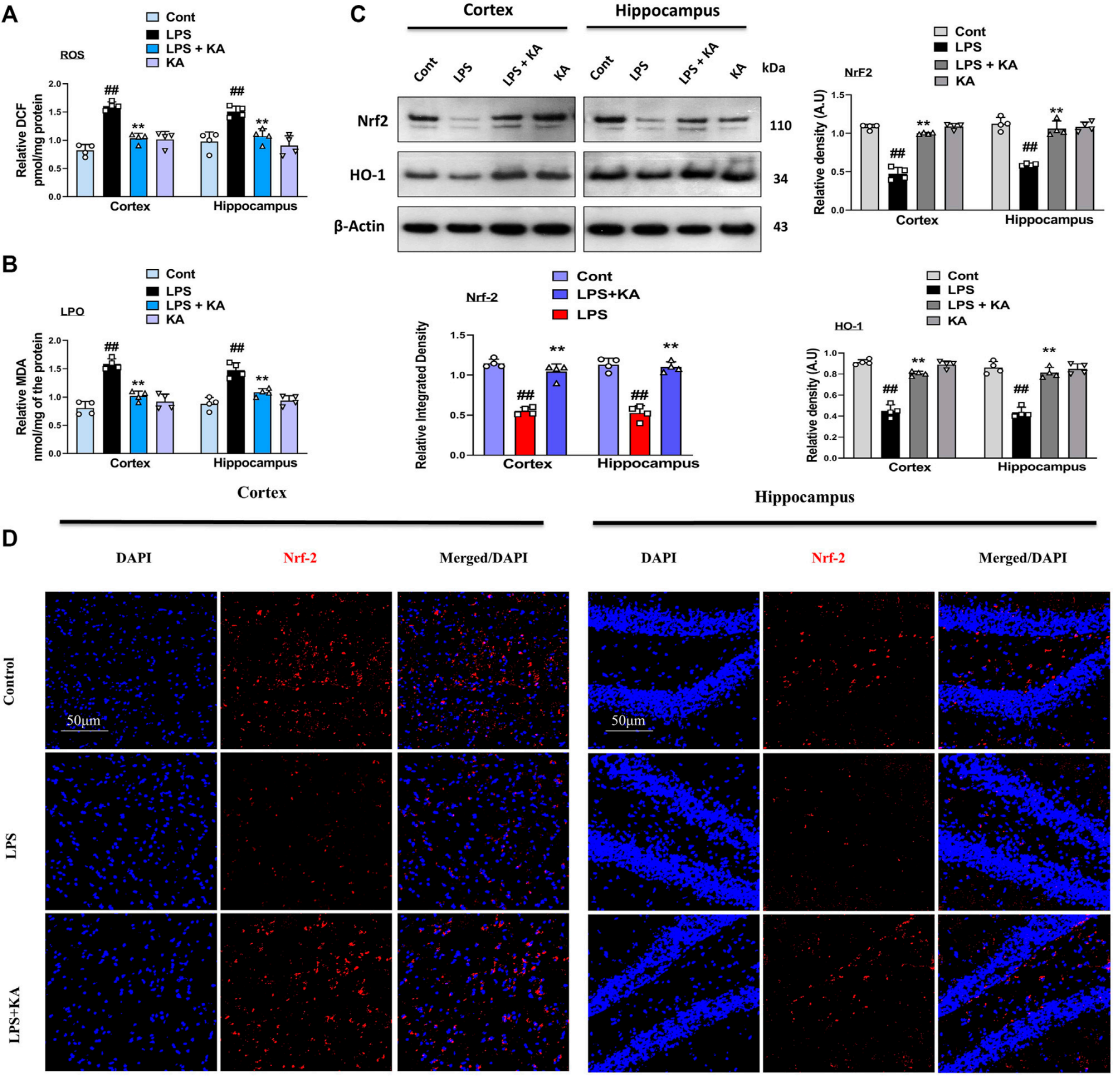
Kojic acid reduced LPS-induced oxidative stress in mouse brain; **(A, B)** reactive oxygen species (ROS) and lipid peroxidation (LPO) assay, respectively; **(C)** immunoblot analysis (n = 4) of Nrf2 and HO-1 protein expression in the mouse cortex and hippocampus; **(D)** immunofluorescence images of Nrf2 in the experimental group; scale bar 50 µm. All the data were measured in mean ± S.E.M. (n = 4), with respective bar graphs. ^#^ significantly different from the saline-injected group, * significantly different from the LPS-injected group. Significance: ^#^ *p* ≤ 0.05, ^##^ *p* ≤ 0.01, and ^###^ *p* ≤ 0.001; *p ≤ 0.05, **p ≤ 0.01, and ***p ≤ 0.001.

Kojic acid exerts an antioxidant effect to prevent oxidative stress-induced diseases. To analyze the possible antioxidant effects of kojic acid against LPS-induced oxidative stress, we evaluated both the expressions of LPO and ROS in the cortex and hippocampus tissue homogenates of the experimental groups. The expressions of LPO and ROS were increased in the LPS-injected mouse group as compared to the saline-injected mouse brain. Interestingly, these effects were significantly reversed in the LPS + kojic acid co-treated group (Figure 4A, B respectively). Complementary biochemical assays such as Western blotting were performed to gain a comprehensive understanding of NRF2 activation and subcellular localization. We evaluated the level of ROS regulators and their downstream signaling (i.e., Nrf2 and HO-1). Our results showed that the levels of Nrf2 and HO-1 were significantly decreased in the LPS mice group and were upregulated in the LPS + kojic acid co-treated group (Figure 4C). Moreover, we performed immunofluorescence microscopy to assess nuclear NRF2 levels in cells under different experimental conditions. This provides spatial information about NRF2 localization within the nucleus. Our immunofluorescence analysis shows a reduction in the relative intensity of nuclear Nrf2 in the LPS-treated mice group, and it was prominently upregulated in the LPS + kojic acid co-treated group (Figure 4D).

### 3.4 Kojic acid may attenuate LPS-induced synaptic dysfunction

**FIGURE 5:**
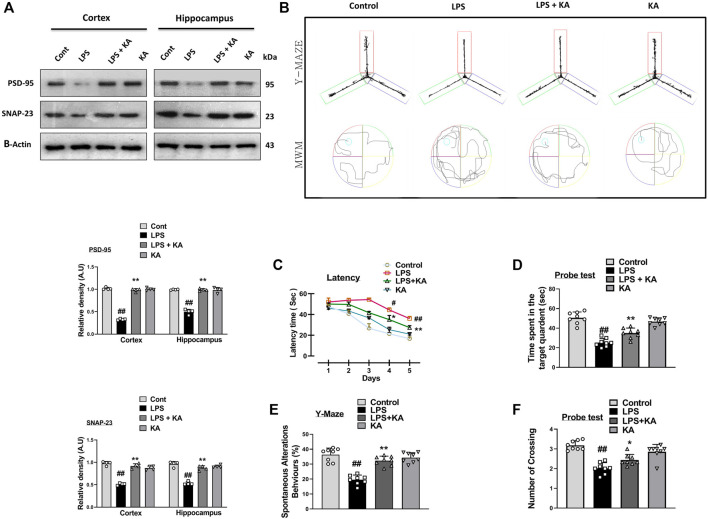
Effect of kojic acid versus LPS-induced synaptic damage and memory impairment. **(A)** Immunoblot results of PSD-95 and SNAP-23 in the cortex and hippocampus of the treated mice groups. **(B)** Y-maze and Morris water maze trajectories. **(C)** Mean time (s) taken to escape during the training days. **(D)** Time spent in the platform quadrant, where the hidden platform was placed during the trial session. **(E)** Spontaneous alteration behavior (%) of the mice during the Y-maze test. **(F)** The average number of crossings at the hidden platform during the probe test of the MWM test. All the data were measured in mean ± S.E.M. (n = 8 per group of animals for behavioral study, n = 4 for Western blotting). ^#^ significantly different from the saline-injected group, * significantly different from the LPS-injected group. Significance: ^#^ *p* ≤ 0.05, ^##^ *p* ≤ 0.01, and ^###^ *p* ≤ 0.001; *p ≤ 0.05, **p ≤ 0.01, and ***p ≤ 0.001.

Various studies have shown that LPS is a very toxic bacterial endotoxin that may affect synaptic integrity (He et al., 2019). Here, we investigated the effect of kojic acid on the synaptic-integrity-related proteins in the LPS-injected mouse brains. For this, we determined the levels of post-synaptic and pre-synaptic biomarkers, such as postsynaptic density protein 95 (PSD-95) and synaptosomal-associated protein 23 (SNAP-23), in different experimental groups. Notably, our Western blot result showed a decrease in the expression of both PSD-95 and SNAP-23 in the LPS-treated mouse brains compared to the normal control mice. Remarkably, these synaptic biomarkers were upregulated in the LPS + kojic acid co-treated group (Figure 5A).

**FIGURE 6:**
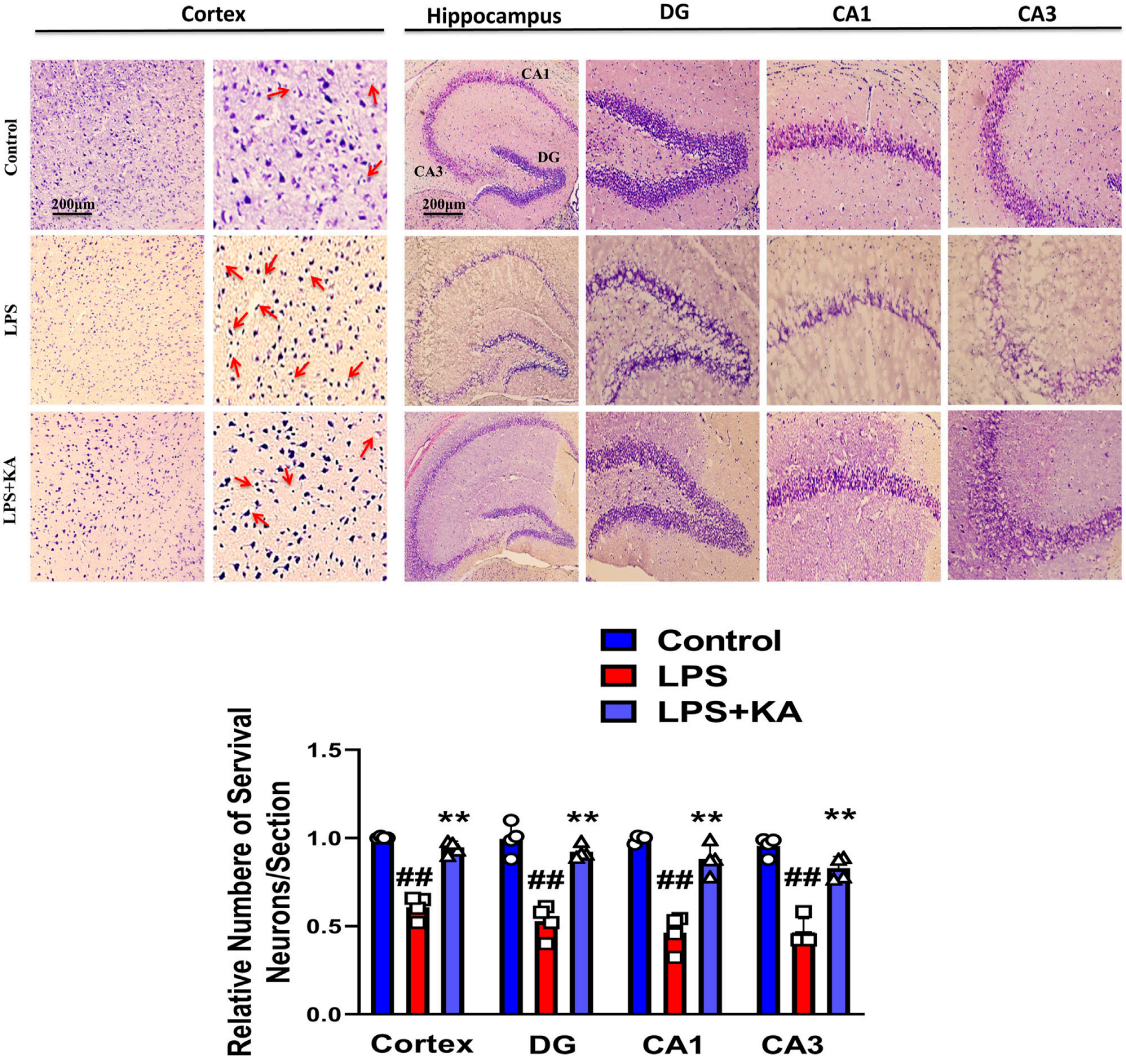
Nissl staining photomicrograph of the DG, CA1, and CA3 regions of the cortex and hippocampus of the mouse brain (n = 4). Scale bar = 200 μm ^#^ significantly different from the saline-injected group, * significantly different from the LPS-injected group. Significance: ^#^ *p* ≤ 0.05, ^##^ *p* ≤ 0.01, and ^###^ *p* ≤ 0.001; *p ≤ 0.05, **p ≤ 0.01, and ***p ≤ 0.001.

We performed the Y-maze and MWM behavior tests to evaluate cognition and memory impairment in rodents. Our Y-maze behavioral test showed short-term memory impairment compared to the normal control. Apart from these, the LPS + kojic acid co-treated mice group improved the percentage of spontaneous alternation behavior and memory impairment (Figure 5B). In the MWM test, the LPS-injected mice took more time to find the hidden platform; this time duration was decreased in LPS + kojic acid-treated mice (Figure 5B). The Nissl staining result showed shrunken, damaged, and fragmented neurons in the cortex and different regions of the hippocampus (i.e., DG, CA1, and CA3) of the LPS-injected mice compared to the saline-treated normal group. The number of shrunken, damaged, and fragmented neurons was much less in the LPS + kojic acid co-treated group, and these brain samples notably retained neuronal cell shape and integrity (Figure 6). Collectively, our results revealed that kojic acid effectively improved synaptic and memory impairments by blocking LPS-induced oxidative stress and neuroinflammation.

## 4 Discussion

The application of natural substances is a primary goal for the prevention and treatment of neurodegenerative diseases. Kojic acid is a bioactive medicinal substance used to slow or prevent neurological disorders. Kojic acid is a well-approved and recommended natural chelating agent that has medicinal properties and protective roles in different paradigms of CNS insult-induced detrimental effects (Liu et al., 2023). In the current study, we investigated the neuroprotective effect of kojic acid against LPS-induced detrimental effects such as neuroinflammation, oxidative stress-mediated neurodegeneration, and synaptic/memory deficits in the cortical and hippocampal regions of the mouse brain.

Neuroinflammation and oxidative stress collectively contribute to neurodegeneration and cognitive impairment. Neurodegenerative diseases (including AD and PD) and their complications affect the patient's quality of life (Adams et al., 2013), reduce self-independence, and may cause death (Mota and Kelly, 2020). The exact cause and risk factors of AD, PD, and other forms of neurodegeneration are still unknown. Different research studies (Zhao et al., 2019) showed that both exogenous (scopolamine, cadmium, and LPS) and endogenous substances (Aβ plaque and p-tau) induced inflammation and oxidative stress, which are responsible for AD-like pathology. Among them, acute and chronic administration of LPS (an endotoxin of Gram-negative bacteria), which binds with toll-like surface receptors expressed on microglia and astrocytes, contributes to neuroinflammation and oxidative stress (Skrzypczak-Wiercioch and Sałat, 2022). TLR4 receptors are also expressed on oligodendrocytes, neurons, and Schwann cells and respond to a wide array of PAMPs and DAMPs, including bacterial, viral, and protozoan substances and heat shock protein (Tang et al., 2023). In response, neuronal loss and glial cell activation lead to downstream signaling pathways through NF-κB, and as a result, various neurotoxic factors and interleukins are released, such as TNF-α and IL-1β (Liu et al., 2017). Similarly, in *in vivo* studies, LPS may change the cognition of study animals, which exhibit symptoms such as decreased locomotion, anxiety, depression, somnolence, decreased appetite, and weight loss, also known as sickness behaviors. Natural compounds like flavonoids, terpenoids, chelating agents, and phenolic substances are considered the main substances to prevent and cure neurodegenerative disorders (Jung et al., 2023). They can inhibit neuroinflammation and oxidative stress and their downstream signaling. Kojic acid is a natural chelating agent (Saeedi et al., 2019) produced and isolated from the fungus *Aspergillus* species. Kojic acid and its derivatives exhibit anti-inflammatory, antioxidant, antiviral, and antifungal properties (Phasha et al., 2022).

It has been previously suggested that activated glial cells lead to increased phosphorylation of NF-κB, which directly plays a major role in neuroinflammation (Khan et al., 2019). The TLR4 receptor is located at the surface of glial cells and responds to various pathogen-derived toxins (LPS) and tissue-damage-associated harmful substances. We analyzed the expression level of TLR4, GFAP, and Iba-1 in the LPS-injected mice brain. Our result suggested that kojic acid inhibits LPS-induced receptor-mediated neuroinflammation in both the cortex and hippocampus, as confirmed with immunoblotting and confocal laser microscopy (Figures 2A, B). Similarly, p-JNK, p-NF-κB, and other inflammatory mediators, including tumor necrosis factor-alpha (TNF-α) and interleukin 1-beta (IL-1β), were increased in the cortex and hippocampus of LPS-injected mice brains (Chuang et al., 2017). Meanwhile, the expression level of these inflammatory biomarkers remarkably decreases in the LPS + KA-treated mice group (Figures 3A, B).

Another parameter that has been considered in the neuroprotective effects of kojic acid is the effects of kojic acid against oxidative stress. Elevated oxidative stress also increases LPO and ROS levels in mice brains (Muhammad et al., 2019). We analyzed the expression level of LPO and ROS in cortex and hippocampus homogenates of mice brains. Notably, our result showed that kojic acid significantly decreases the expression of LPO and ROS (Figures 4A, B). We determined the oxidative biomarkers (Nrf2 and HO-1) in the brains of experimental rodents. Both the Western blot and confocal laser microscopy studies suggested that orally administered kojic acid has a strong antioxidant effect and increased the expression level of Nrf2 and HO-1 (Figures 4C, D), as previous studies suggested that the level of Nrf2 and HO-1 were decreased in LPS-injected mice group.

Neurons consist of synapses and dendrites through which neurons communicate. These synapses and dendritic spines are prone to neurodegenerative diseases (Thingore et al., 2021). Usually, cognitive dysfunction in AD is co-related with synaptic impairment and changes in the number and shapes of dendrites with no neuronal death (Chugh et al., 2013). Different therapeutic agents have been investigated for learning and memory function in various detrimental conditions. Here, the Morris water maze and Y-maze tests were carried out to determine whether KA reverses the cognitive impairments in LPS-treated mice. The learning behavior and spontaneous alternation improved in the LPS + KA mice group compared to the LPS-only-treated mice group. These findings suggest that KA could heal short-term memory loss in the LPS-injected mice group (Figures 5C–F).

Several studies have shown that LPS-induced neuroinflammation and oxidative stress mediate synaptic and memory dysfunction (Bilbo et al., 2005). Our results suggest that kojic acid treatment increased synaptic integrity by enhancing synaptic protein in the cortex and hippocampus in the LPS-injected AD mouse model (Figure 5A).

The Nissl staining results support our data, and the LPS-injected group brain showed neuronal cell shrinkage, damage, and fragmentation in both the cortex and the hippocampus (DG, CA1, & CA3). This damage was much less apparent in the LPS + KA-treated mice group.

## 5 Conclusion

**FIGURE 7:**
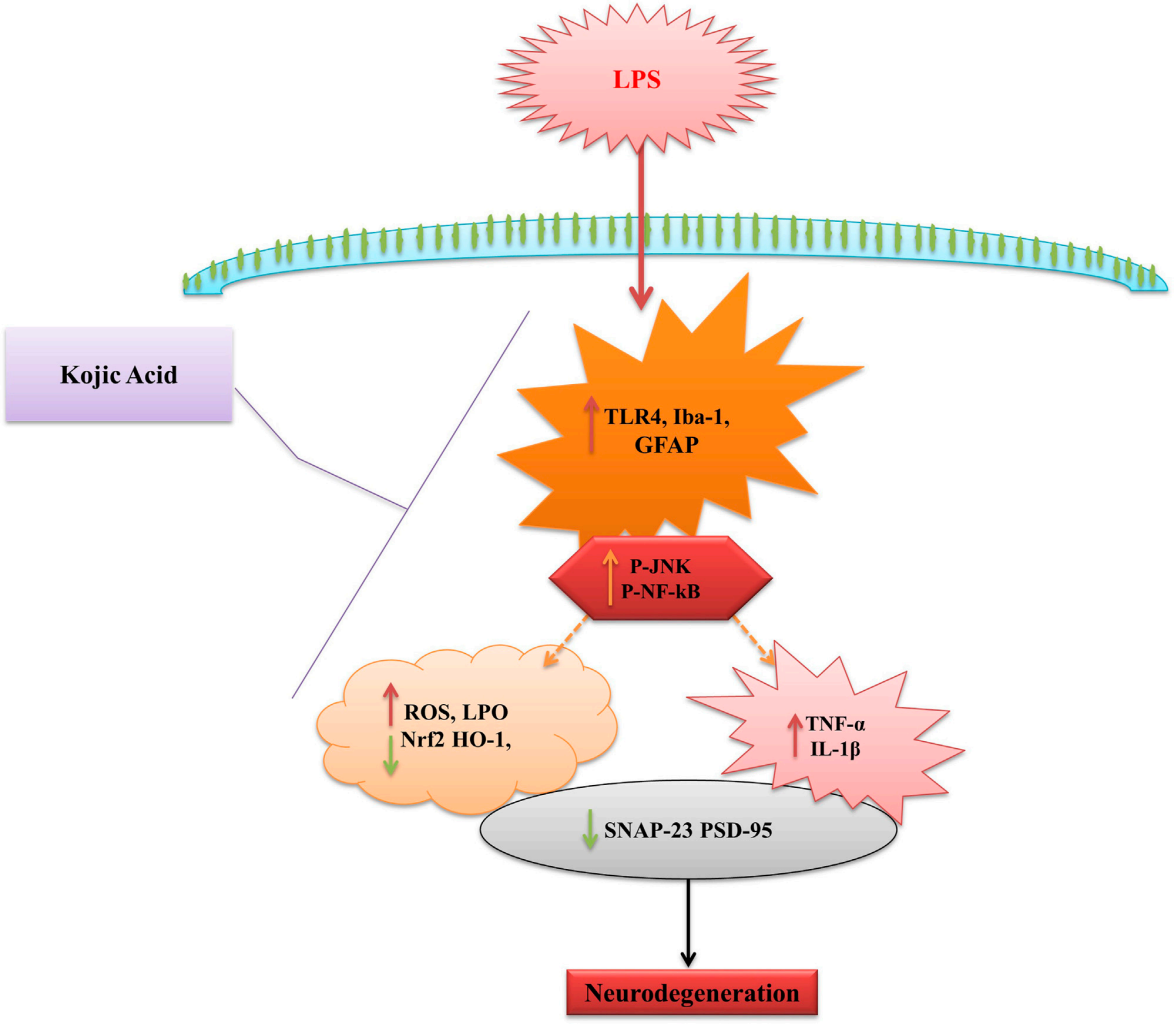
Schematic diagram represents the proposed mechanism of neuroprotection by KA against LPS-induced neuroinflammation, oxidative stress, and cognitive dysfunction in the mouse brain.

In conclusion, kojic acid abrogated the inflammatory responses and oxidative stress in male C57BL/6N mice. Kojic acid regulates the TLR4 surface receptors of microglia and astrocytes. Additionally, we showed that kojic acid suppresses the pro-inflammatory response via downregulation of NF-κB activation. Notably, our result also demonstrated that kojic acid blocks the secretion of TNF and IL-1β by macrophage cells. Furthermore, our result suggested that kojic acid may act as a bioactive natural compound with both anti-inflammatory and antioxidant activity and may play a major role in curing inflammatory and oxidative stress-induced diseases (Figure 7).

## Data Availability

The original contributions presented in the study are included in the article/supplementary material; further inquiries can be directed to the corresponding authors.
